# Proteomic-based biotyping reveals hidden diversity within a microalgae culture collection: An example using *Dunaliella*

**DOI:** 10.1038/srep10036

**Published:** 2015-05-12

**Authors:** Kaveh Emami, Ethan Hack, Andrew Nelson, Chelsea M. Brain, Fern M. Lyne, Ehsan Mesbahi, John G. Day, Gary S. Caldwell

**Affiliations:** 1 School of Marine Science and Technology, Newcastle University, Newcastle upon Tyne, NE1 7RU, UK; 2 School of Biology, Newcastle University, Newcastle upon Tyne, NE1 7RU, UK.; 3 Faculty of Health and Life Sciences, Northumbria University, Newcastle upon Tyne, NE1 8ST; 4 Faculty of Science, Agriculture and Engineering (SAgE), Devonshire Building, Newcastle University, Newcastle upon Tyne, NE1 7RU, UK; 5 Culture Collection of Algae and Protozoa, Scottish Association for Marine Science, Scottish Marine Institute, Oban, Argyll, PA37 1QA, UK

## Abstract

Accurate and defendable taxonomic identification of microalgae strains is vital for culture collections, industry and academia; particularly when addressing issues of intellectual property. We demonstrate the remarkable effectiveness of Matrix Assisted Laser Desorption Ionisation Time of Flight Mass Spectrometry (MALDI-TOF-MS) biotyping to deliver rapid and accurate strain separation, even in situations where standard molecular tools prove ineffective. Highly distinctive MALDI spectra were obtained for thirty two biotechnologically interesting *Dunaliella* strains plus strains of *Arthrospira*, *Chlorella*, *Isochrysis, Tetraselmis* and a range of culturable co-occurring bacteria. Spectra were directly compared with genomic DNA sequences (internal transcribed spacer, ITS). Within individual *Dunaliella* isolates MALDI discriminated between strains with identical ITS sequences, thereby emphasising and enhancing knowledge of the diversity within microalgae culture collections. Further, MALDI spectra did not vary with culture age or growth stage during the course of the experiment; therefore MALDI presents stable and accurate strain-specific signature spectra. Bacterial contamination did not affect MALDI’s discriminating power. Biotyping by MALDI-TOF-MS will prove effective in situations wherein precise strain identification is vital, for example in cases involving intellectual property disputes and in monitoring and safeguarding biosecurity. MALDI should be accepted as a biotyping tool to complement and enhance standard molecular taxonomy for microalgae.

The search for, and exploitation of marine and aquatic organisms for biotechnology applications - so called blue biotechnology^1^ - has risen to the forefront of the global research agenda over the past decade. Algae and cyanobacteria are intrinsic to the success of this sector^2^,^3^ and phycological research has benefitted greatly from the buoyant funding landscape this has created. Algae and cyanobacteria are some of the main biological protagonists that drive many global biogeochemical cycles, being directly responsible for over half of current planetary productivity and geologically for many fossil-fuel resources^4^. Their high conversion of solar energy into biomass, capacity to grow in saline or hypersaline environments, ability to metabolise industrial and domestic waste streams (including CO_2_ flue gases and wastewater), combined with the diverse and arguably untapped range of biochemicals they can synthesise^5^,^6^ makes algae and cyanobacteria attractive bioprospecting targets^7^,^8^. In this regard, culture collections that curate microalgae and cyanobacteria, whether public or private, have proven to be fruitful and convenient biological reservoirs for bioprospecting^8^,^9^.

Industry interests, often focused on intellectual property rights, are core to the boom in blue biotechnology^10^,^11^ with investment strategies often hinging on protection of novel or high performing strains, despite the fact that legal issues remain regarding the patenting of microbes in their wild-type versus genetically modified or even synthetic forms^12^,^13^. Added to this are concerns relating to biosecurity and traceability of industrial or genetically transformed strains, particularly if grown in open outdoor culture systems or in the event of an unwanted environmental discharge^14^. With this in mind, the need for rapid, accurate and defendable taxonomic identification of microalgae and cyanobacteria strains is paramount for culture collections, industry and academia; particularly when addressing issues of intellectual property and biosecurity.

A variety of approaches have been applied, successfully and otherwise, to characterise microalgae and cyanobacteria; including morphology and ultrastructure studies, genetic studies such as examining the secondary structures of the small subunit ribosomal RNA, the internal transcribed spacer (ITS) region of ribosomal DNA, Amplified Fragment Length Polymorphisms and metagenomics^15^,^16^,^17^,^18^,^19^. It has been estimated that there are well in excess of one thousand species of green algae alone^20^. *Dunaliella* species produce biochemicals with applications ranging from pharmaceuticals, high value pigments, and biofuels and platform chemicals^21^,^22^,^23^,^24^,^25^. Although the genus *Dunaliella* was introduced in 1905^26^, due to the ecological, morphological and biochemical plasticity of this alga (particularly in relation to cell size, pigmentation and other nominally defining characters) the taxonomy of the genus has not been clearly resolved^17^. However, genetic barcoding approaches have identified twenty eight species separated into two main ecological groups, freshwater and marine species, with both groups having their own subdivisions^27^,^28^,^29^,^30^,^31^. In general, ITS rDNA sequencing has been accepted as a powerful tool for distinguishing microalgae species^32^ although the *tuf*A gene has recently been advanced as a more appropriate DNA biomarker for chlorophytes^18^. ITS biotyping has been used for phylogeny of *Dunaliella* strains by several investigators^15^,^33^,^34^, with the most recent and comprehensive work undertaken by Assunçäo and co-investigators^27^,^35^ using ITS2. Analysis of ITS1, ITS2 and ITS2 secondary structure has revealed high intraspecific variation within *Dunaliella*^15^,^33^,^34^,^36^,^37^. ITS2 is thought to be an excellent marker for molecular phylogenetic studies, especially at lower taxonomic levels^38^.

An alternative approach to DNA-based strain separation that has very rarely been applied to microalgae and cyanobacteria is proteomic-based chemotaxonomic biotyping using MALDI-TOF mass spectrometry. Using mass spectrometers for characterising microorganisms began in 1975^39^. Thereafter, the methods were rapidly optimised and expanded to include clinical and environmental species. MALDI-TOF-MS biotyping has successfully been used to identify bacteria, yeast, fungi, and even higher eukaryotes including insects^40^, molluscs^41^ and fish^42^. It has also been used in clinical and environmental contexts to identify and differentiate between strains of the pathogenic, microalga *Prototheca*^43^,^44^ and the bloom forming cyanobacterium *Oscillatoria*^45^. However, the potential for the approach to be applied to blue biotechnology has not hitherto been explored. As the molecular basis for the differences in MALDI-TOF-MS spectra are generally unknown the differences cannot be assumed to reflect phylogeny. The power of the method is in the potential capacity to discriminate between closely related organisms. In this context, we have undertaken a rigorous evaluation of the capacity of biotyping by MALDI-TOF-MS to function as both a complementary and standalone tool to differentiate between closely related strains of the commercially important chlorophyte microalgal genus *Dunaliella*.

## Results

### ITS sequence analysis

**Figure 1:**
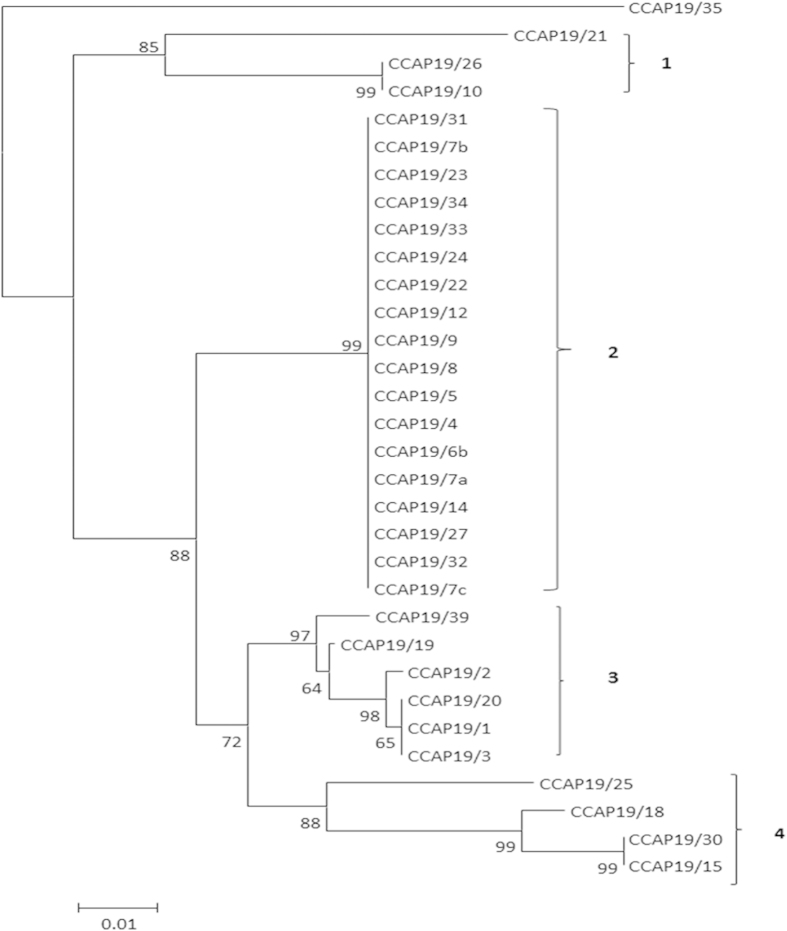
ITS phylogram of all thirty two *Dunaliella* isolates identified by strain number (see Table 1 for assigned species names) available from the Culture Collection of Algae and Protozoa (CCAP). Four distinctive phylogenetic clades, labelled 1-4, were identified containing between three and eighteen isolates. The exception was *D. acidophila* that had no significant nucleotide similarity with any other CCAP isolate.

The thirty two *Dunaliella* isolates investigated here were previously classified and deposited with the Culture Collection of Algae and Protozoa (CCAP) on the basis of their gross phenotype (cell shape and size) and, in some cases using limited biochemical properties such as carotenoid production. In this study by using ITS4 and ITS5 primers, 729 bp of rDNA including ITS1, ITS2, and the 5.8S rRNA gene were amplified and sequenced. Three isolates, CCAP 19/10, 19/21, and 19/26, contained introns in their sequences. The introns were excluded during the construction of the phylogram presented in Fig. 1. Four major clades were observed encompassing thirty one of the thirty two available strains. The one exception was *D. acidophila* (CCAP 19/35), which stands as a distinctive strain. Clade 1 consisted of two strains of *D. parva* (CCAP 19/10 and 19/26) and one strain (CCAP 19/21) not previously designated to species level. Whilst not included in the phylogram construction, CCAP 19/10 and 19/26 have identical introns that imply that they are isolates of the same species. Clade 2 comprised eighteen isolates of which six have not previously been designated to species level. The remaining twelve isolates have been classified as eight distinct species (Table 1). In contrast, clade 2 is the only clade in which all isolates have identical ITS sequences (Fig. 1). Clade 3 consisted of three *D. salina,* one *D. parva,* one *D. peircei* isolates and a strain not previously designated to species level. Clade 4 contained three strains deposited as *D. salina* and one strain not previously designated to species level. Further analysis of the ITS2 sequence and structure supports the finding of the sequence only tree (Supplementary Figure 1).

### MALDI-TOF analysis of microalgae and cyanobacteria isolates

A twenty day time point experiment using CCAP 19/7C and 19/27 demonstrated no significant difference between the general spectral pattern of the two isolates with time despite the cultures having been sampled during phases of logarithmic and linear growth, early and late stationary growth phases and early culture senescence (see Supplementary Figure 2). The only differences observed were in the intensity of the major peaks rather than their mass to charge ratio by MALDI-TOF. The MALDI-TOF-MS biotyping approach was therefore applicable throughout the full culture cycle.

Good quality spectra were obtained for numerous bacterial species that were co-occurring with two *D. polymorpha* isolates, two of which are presented in supplementary Fig. 4. There was no major overlap of peaks when overlaid with the spectra for *D. polymorpha*. Similarly, treatment of the cultures with antibiotics did not significantly change the *D. polymorpha* spectra.

**Figure 2:**
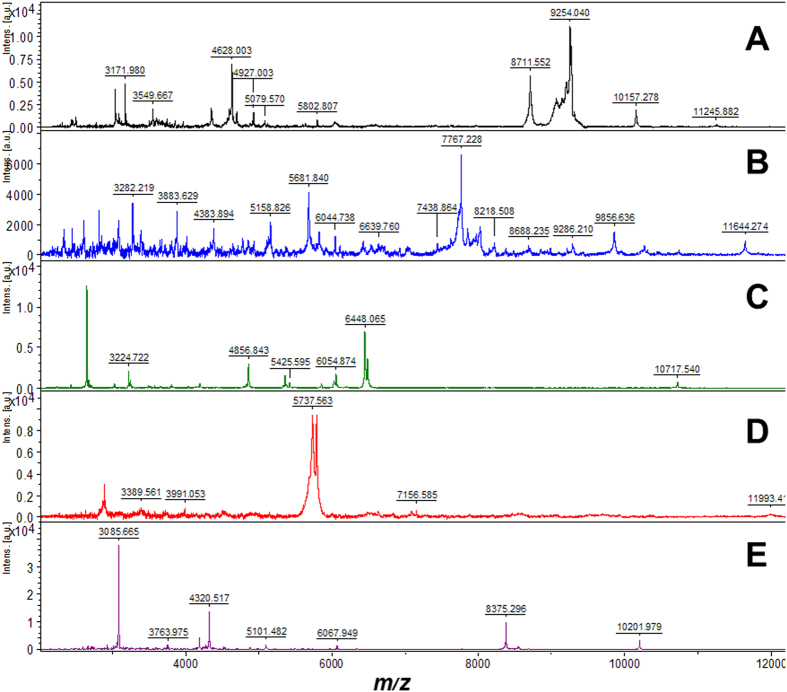
Comparison of the MALDI-TOF mass spectra of: **A**) *Dunaliella polymorpha* (CCAP 19/7B); **B**) *Arthrospira platensis* (CCMP 1295); **C**) *Chlorella vulgaris* (CCAP 211/63); **D**) *Isochrysis galbana* (CCAP 927/1); and **E**) *Tetraselmis suecica* (Seasalter Shellfish Ltd). Each mass spectrum was markedly different demonstrating that biotyping by MALDI-TOF-MS can easily distinguish between microalgae and cyanobacteria genera.

An initial experiment was conducted to validate the efficacy of biotyping by MALDI-TOF-MS to discriminate between distantly related microalgae and cyanobacteria genera using four eukaryote species; the chlorophytes *Dunaliella polymorpha*, *Chlorella vulgaris* and *Tetraselmis suecica*, the haptophyte *Isochrysis galbana*; and one prokaryote species; the cyanophyte *Arthrospira platensis* (Fig. 2). Each tested species generated highly distinct mass spectra, therefore demonstrating little chance of spectral overlap between separate genera.

**Figure 3:**
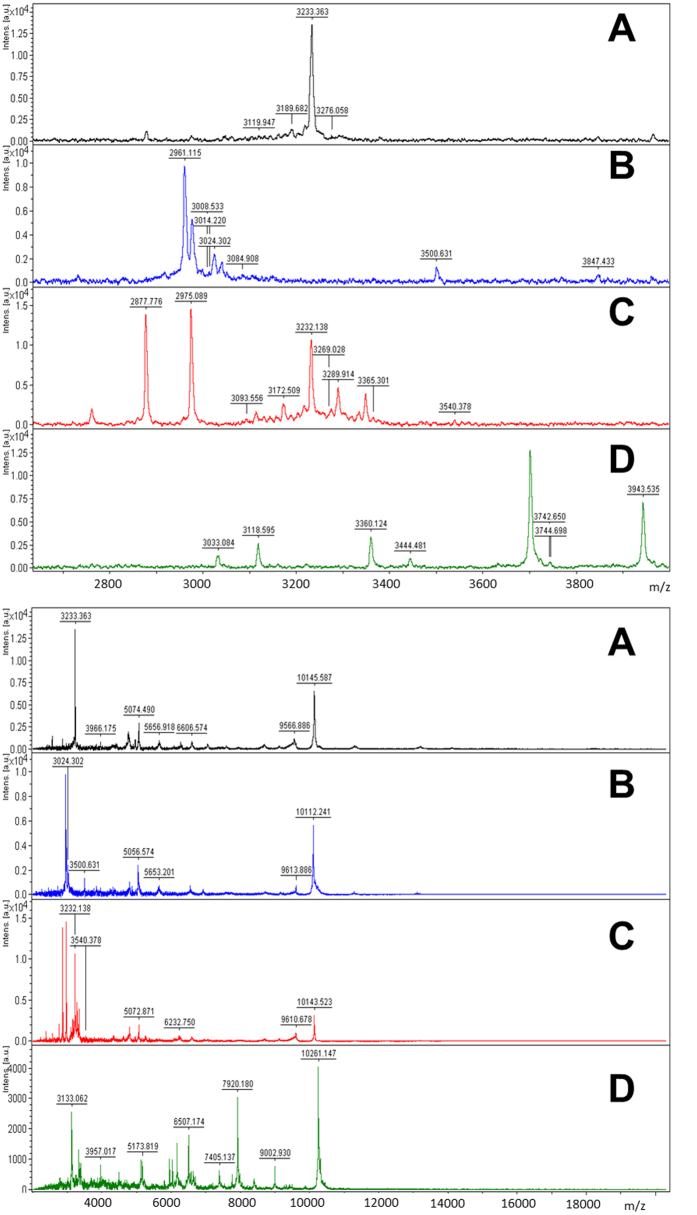
MALDI-TOF mass spectra of the three *Dunaliella* isolates assigned to clade 1 in the ITS rDNA dendrogram (Fig. 1): **A**) CCAP 19/10; **B**) CCAP 19/21; and **C**) CCAP 19/26. Additionally, the spectrum for *D. acidophila* (CCAP 19/35) which had no significant ITS nucleotide sequence similarity with any other CCAP isolate is presented in panel D. Peaks in the *m/z* range of 2620-3500 for CCAP 19/10, 19/21 and 19/26 and *m/z* 2600-4000 for CCAP 19/35 are presented in the upper panels and peaks in the *m/z* range of 2000-15000 are presented in the lower panels. CCAP 19/10 and 19/26 isolates were deposited as *D. parva* and have identical ITS rDNA sequences yet the peaks at *m/z* 2975 and 3289 in CCAP 19/26 are absent in CCAP 19/10 and 21. However the peak at *m/z* 2961 is only present in the CCAP 19/21 spectrum. CCAP 19/10 and 19/26 also have common peaks at *m/z* 2877 and 3232.

To determine whether biotyping by MALDI-TOF-MS may provide enhanced between-strain discrimination compared with ITS sequencing alone, the MALDI spectra of each ITS generated clade were analysed. MALDI-TOF-MS biotyping confirmed the output of ITS sequencing highlighting that *D. acidophila* (CCAP 19/35) is the most distantly related of the CCAP *Dunaliella* isolates. The spectrum is presented in Fig. 3 alongside clade 1 (CCAP 19/10, 19/21 and 19/26) for comparison. CCAP 19/21 has not previously been identified to species level, whereas both CCAP 19/10 and 19/26 were deposited as *D. parva* and their ITS rDNA sequences are identical. Peaks at *m/z* 2878, 3233, 5072, 6606, 10143 and 13211 are common between the *D. parva* isolates which suggests that they are indeed the same species. Their mass spectra also show strong similarities to that of CCAP 19/21. CCAP 19/21 and 19/26 have a peak at *m/z* 9611 ± 2, whereas CCAP 19/10 and 19/21 have a peak at *m/z* 5654 ± 2.

Of particular interest were the mass spectra (summarised in Table 2) corresponding to the eighteen isolates comprising clade 2 that yielded identical ITS rDNA sequences. Nine major peaks were identified within clade 2 occurring at *m/z* 3045, 4627, 5803, 7588, 8711, 9254, 10157, 13019 and 18101 respectively. The mass spectra of eight of the isolates (CCAP 19/4, 9/7A, 19/7B, 19/7C, 19/8, 19/14, 19/22 and 19/24) were almost identical, each sharing seven of the nine major peaks but missing peaks at *m/z* 7588 and 18101. However, CCAP 19/8 was missing a peak at *m/z* 6473 which was present in the other isolates (data not shown). Both rDNA and MALDI results suggest these strains belong to the same *Dunaliella* species yet the current CCAP nomenclature places CCAP 19/7A, 19/7B, and 19/7C as *D. polymorpha*, CCAP 19/4 as *D. bioculata*, CCAP 19/8 as *D. quartolecta*, CCAP 19/24 as *D. tertiolecta* and CCAP 19/14 and 19/22 as *Dunaliella* sp. CCAP 19/23 (*Dunaliella* sp.) and 19/31 (*D. salina*) also had identical spectra sharing seven major peaks but missing the peaks at *m/z* 5803 and 18101. The remaining eight isolates were each distinct from all other members of the clade.

**Figure 4:**
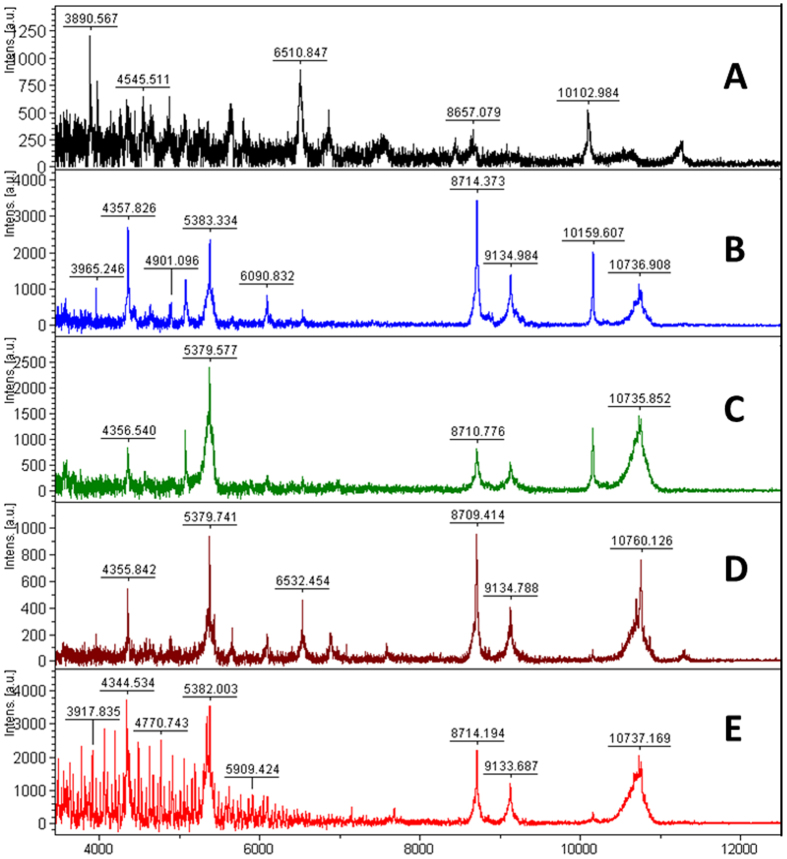
MALDI-TOF mass spectra of five of the six *Dunaliella* isolates assigned to clade 3 in the ITS rDNA dendrogram (Fig. 1): **A**) CCAP 19/1; **B**) CCAP 19/2; **C**) CCAP 19/3; **D**) CCAP 19/19; and **E**) CCAP 19/20. The spectra for CCAP 19/20 contained a polymeric pattern similar to that of CCAP 19/18 (Fig. 5) but did not interfere with spectral analysis. The spectra for CCAP 19/39 was of lower presentational quality and is omitted.

Clade 3 of the ITS phylogram includes CCAP 19/1 (*D. parva*), 19/2 (*D. peircei*), 19/3 (*D. salina*), and 19/20 (*D. salina*) with very similar ITS rDNA, also CCAP 19/19 (*Dunaliella* sp.) and 19/39 (*D. salina*). Mass spectra of these isolates are compared in Fig. 4. Although CCAP 19/1 did not generate a high quality sectrum, the MALDI fingerprint has marked similarities to the other strains in this clade and was included in the analysis. The CCAP 19/20 mass spectrum contained a polymeric pattern that did not interfere with clustering and resolution of the mass spectra, with peaks gap of *m/z* 142. Peaks at *m/z* 4357, 5380, 8714, 9134, 10735 were observed in most isolates.

**Figure 5:**
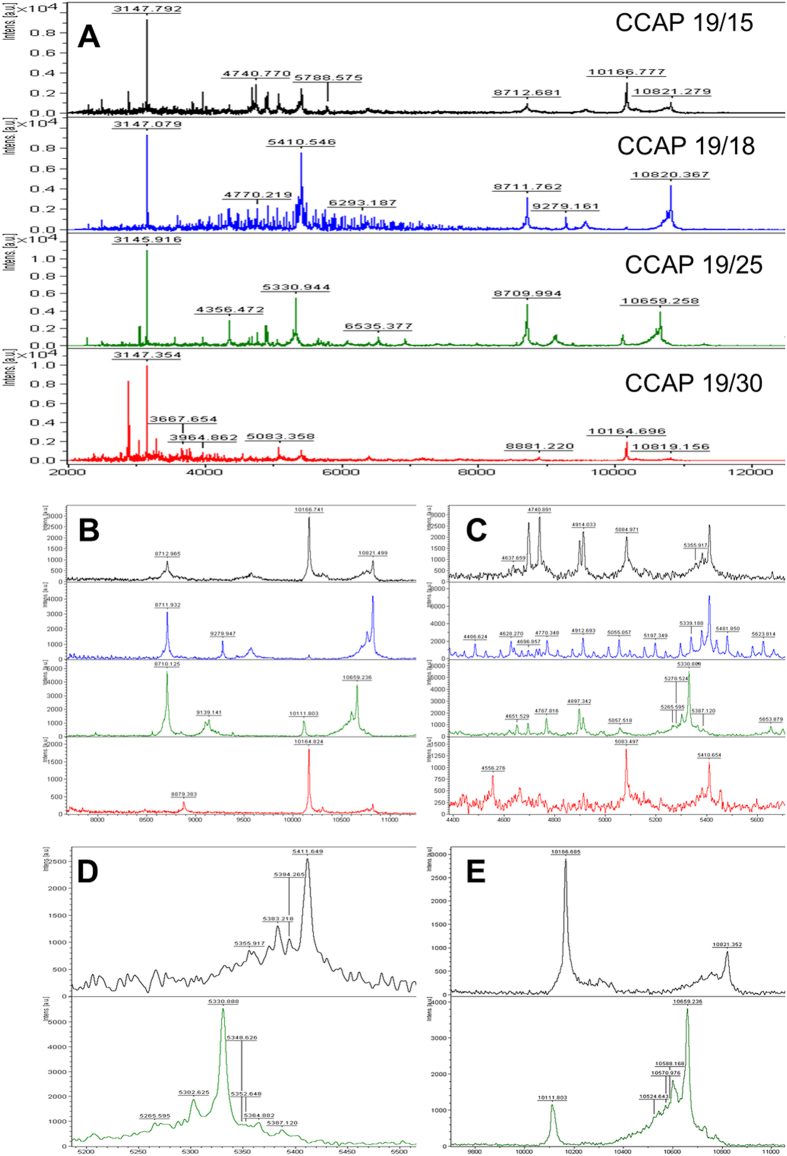
MALDI-TOF mass spectra of the four *Dunaliella* isolates assigned to clade 4 in the ITS rDNA dendrogram (Fig. 1). Most peaks had very similar mass to charge ratios. Panel A shows the mass spectra between *m/z* 2400-12200, the region of the spectra where most peaks appeared. Panels B (*m/z* 8000-12000) and C (*m/z* 3500-6000) present more detailed examples of similar and distinctive peaks between the four isolates. Panels D (*m/z* 5200-5700) and E (*m/z* 9000-11400) show examples of peak differences between CCAP19/15 and CCAP 19/25.

Clade 4 of the ITS phylogram contains three *D. salina* strains (CCAP 19/18, 19/25 and 19/30) and one strain (CCAP 19/15) not previously designated to species level. CCAP 19/15 and 19/30 have identical ITS sequences and their mass spectra are also very similar (Fig. 5), strongly indicating that both isolates are the same species. All the isolates in this clade have peaks at approximately *m/z* 3146, 3965, 5411, and 8711 (Fig. 5A,B). On this basis CCAP 19/15 is very likely *D. salina*. In this clade CCAP 19/25 possessed the most distinct fingerprint. The other three isolates had peaks at *m/z* 10166 ± 1 but CCAP 19/25 lacked this peak, instead it had unique peaks at *m/z* 9139, 10659 and 13489 (Fig. 5B-E). CCAP 19/18 yielded a polymer-type pattern similar to that observed from CCAP 19/20; again, the polymer did not interfere with spectral resolution.

**Figure 6:**
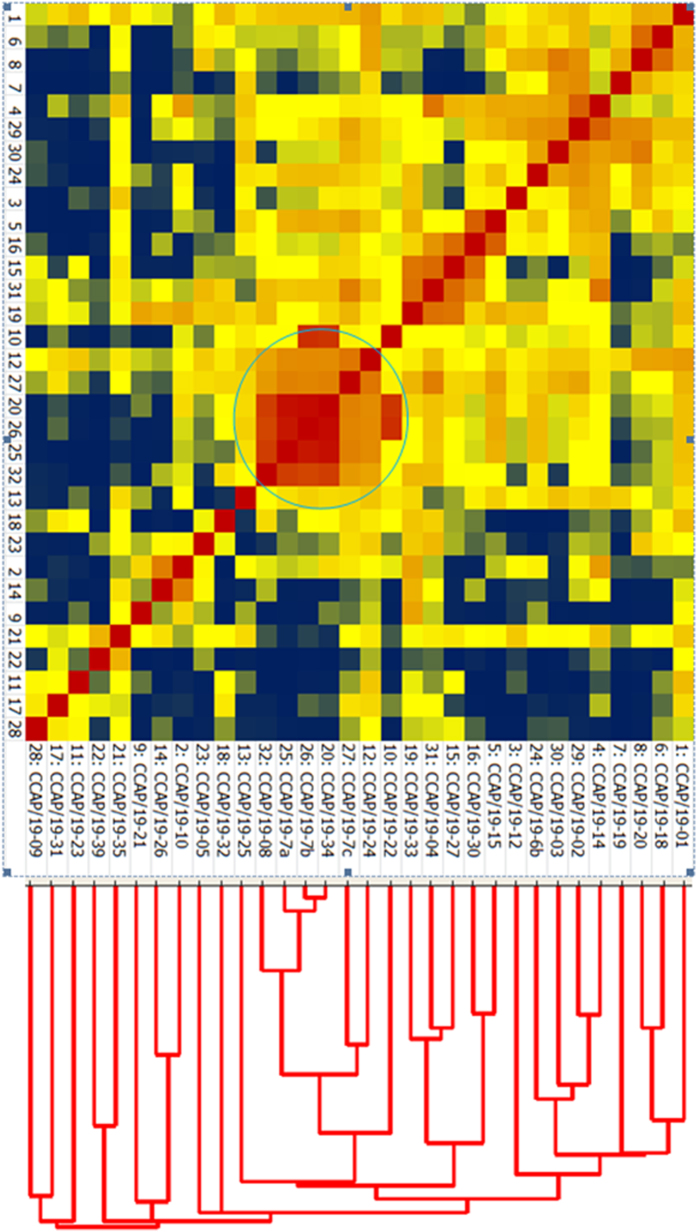
Composite correlation index based heat map and corresponding dendrogram generated from MALDI-TOF analysis for all thirty two CCAP *Dunaliella* isolates. Red colouration represents the highest degree of similarity, yellow corresponds to the 50^th^ percentile of similarity, and blue represents the lowest similarity. Isolates with the most similar spectra are circled. Numbers on the bottom of the heat map correspond to those on the side. It is clear that biotyping by MALDI-TOF-MS has a greater strain resolving power than ITS rDNA sequencing (Fig. 1) alone.

An overall view of the relatedness of the CCAP *Dunaliella* isolates based on MALDI mass spectral patterns is presented in Fig. 6 in both dendrogram and heat map formats. The most closely related isolates are indicated by red squares and the least in blue. The isolates CCAP 19/8, 19/24, 19/34, 19/7A, 19/7B, and 19/7C are at the core of the heat map due to the high degree of similarity across their spectra. Heat map mass spectral clustering of the isolates CCAP 19/10, 19/21 and 19/26 concurs with the ITS sequencing confirming that these isolates are closely related (Figs. 1 and 6).

## Discussion

The use of ITS sequences to resolve microalgae taxonomy to species level is widely accepted although the *tufA* plastid gene sequence would appear to provide greater resolving power for the green algae^18^. Based on crossing experiments, it has been proposed that even a single Compensatory Base Change (CBC) in helices 2 and 3 of the ITS2 indicates sexual incompatibility and thus separate biological species^32^. Furthermore, secondary structure analysis can improve the phylogenetic resolution obtained from the primary sequence^46^.

*Dunaliella* species share very high ITS sequence similarity and similar phenotypic traits^15^,^17^,^27^,^33^,^34^,^35^,^36^,^37^; therefore, taxonomic definition of *Dunaliella* species requires a thorough understanding of the molecular and biochemical characteristics of the isolates. The current taxonomic designation of the thirty two CCAP isolates used in this study (Table 1) follows the nomenclature of the original depositor and was based primarily on a morphological approach. DNA barcoding has not previously been employed to re-evaluate these strains. The ITS phylogeny assigned thirty one of the thirty two strains to one of four main clades, the exception being CCAP19/35 *D. acidophila* which was clearly distinct from all other strains. This is supported by the requirement for this strain to be held in highly acidic conditions to allow growth whereas the other strains have pH optima closer to that of ambient seawater. Clade 2 was particularly interesting as all eighteen isolates had identical ITS sequences yet twelve of these strains have been assigned to eight separate species based on morphological traits, with the remaining six isolates as yet not classified to species level. Clearly there is a discrepancy between classical taxonomic approaches and ITS barcoding. Due to the high level of DNA sequence similarity the value of ITS2 structure analysis is limited for the differentiation of morphologically distinct isolates (Supplementary Fig. 1). If the barcoding approach is accurate then this serves to highlight the high degree of morphological plasticity exhibited by this genus.

Although the most reliable standard in algal taxonomy remains DNA-DNA hybridisation among species, it is not always feasible to apply this method to all isolates of interest^18^,^47^. Furthermore, as algal biotechnology continues to develop there is already a growing ground swell within the community aimed towards deploying mutant and transgenic strains; with the concomitant absolute requirement for methods capable of identifying biochemically distinct environmental isolates. Alarmingly, standard molecular barcoding methods are not capable of this level of resolution. There are suggestions that whole genome sequence data may eventually replace DNA-DNA hybridisation approaches^48^ and this would certainly provide the level of definition required to differentiate algae at species or even strain level. However, cost implications, particularly with respect to data assembly, interpretation and quality assurance still make this impracticable in the short to medium term.

In this study we have aimed to explore the utility of MALDI-TOF-MS biotyping as a method to bridge this gap between ITS based DNA barcoding and whole genome sequencing for the identification and differentiation of *Dunaliella* isolates. Chemotaxonomic techniques such as MALDI-TOF-MS biotyping are now commonly used in many microbiology laboratories worldwide and are capable of detecting very minor differences in the expression of major ribosomal and housekeeping genes. The method has been expanded to the identification and characterisation of eukaryotes^40^,^42^,^43^,^44^; yet it remains an underutilised technique in the study of microalgae, particularly from a biotechnology perspective^49^. Figure 3 clearly shows that the approach can easily distinguish between genera, yet the real perceived value of the approach lies with its potential to differentiate between isolates within the same genus and ideally within the same species. The target of the current study was to assess the feasibility of biotyping by MALDI-TOF-MS to discriminate between strains of *Dunaliella* held in the publically accessible CCAP collection ( http://www.ccap.ac.uk/). However, the authors support the assertion that sexual compatibility remains the gold standard for the phylogeny of this genus.

In clade 1 of the ITS phylogram CCAP 19/10 and 19/26 have identical ITS sequences and they share MALDI peaks at *m/z* 5074 ± 2 and 10144 ± 1; however, clustering based on MALDI data (Fig. 6) suggests that the strains are distinct. The third strain in clade 1, CCAP 19/21 has a mass difference in the two main peaks. The MALDI data therefore confirms that all three strains cluster together but also highlights differences between the strains. Interestingly, the MALDI analysis for *D. acidophila* CCAP 19/35 suggests it may not be entirely distinct from all other strains. There appeared to be some commonality with *D. salina* CCAP 19/39; however, this is not a strong indicator of relatedness. For clade 1 the ITS and MALDI approaches are generally consistent.

For clade 3 again there is broad agreement between the two approaches. Clade 4 tells a similar story with two strains, CCAP 19/30 and 19/15 in very close alignment across both techniques, yet MALDI suggests that CCAP 19/18 and 19/25 do not cluster with each other or either of the two clade 4 strains.

The most interesting differences are highlighted for the strains within clade 2, all of which have identical ITS sequences. Biotyping by MALDI-TOF-MS would suggest that four separate clusters are identifiable within this clade; 1) CCAP 19/14, 19/6B and 19/12; 2) CCAP 19/32 and 19/5; 3) CCAP 19/23, 19/31 and 19/9; and finally 4) CCAP 19/27, 19/4, 19/33, 19/22, 19/24, 19/7C, 19/34, 19/7B, 19/7A and 19/8. Clustering is based on the presence and absence of indicator peaks and also changes in peak mass to charge ratio (Table 2).

One concern of the MALDI approach is the presence and potentially confounding influence of bacteria or other microbial contaminants within the algae. As most algae cultures are non-axenic and in some cases there is an absolute requirement for bacteria-algae interactions to ensure normal healthy growth^50^, this may restrict the applicability of the method. If biotyping by MALDI-TOF-MS was found to only be applicable to axenic cultures, this would necessitate the addition of antibiotics to the cultures. None of the cultures used in the study were axenic; therefore, there is a risk that contaminants may potentially interfere with the acquisition of a pure algae mass spectra. Indeed, further investigation revealed that the polymer-like patterns observed from CCAP 19/18 and 19/20 were due to the presence of culturable bacteria (data not shown); although the bacterial MALDI-TOF spectrum did not share any common peaks with those of the respective *Dunaliella* isolates. Similarly, the spectra obtained from numerous bacteria isolated from two cultures of *D. polymorpha* (supplementary Figure 4; manuscript in preparation) did not overlap with the microalgae peaks. Indeed, the bacterial biomass relative to the algae biomass within the *D. polymorpha* cultures was too low to make any real impact on the alga’s spectral fingerprint. Antibiotic treatment of cultures supported this conclusion (data not shown).

There are contradictory reports as to the effect of culture media and culture age on bacterial protein mass-spectral patterns. Pennanec *et al.*^51^ and Ruelle *et al.*^52^ found no significant change in mass spectra fingerprint which may be explained by the dominance of peaks resulting from ribosomal and house-keeping proteins which generally remain unchanged under different growth conditions and throughout different growth stages^53^,^54^,^55^. However, Valentine *et al.*^56^ observed significant differences in the mass spectra of bacteria when grown on different media. Similarly, Salaün and coinvestigators^57^ noted that culture media did affect the spectral signature in bacteria but culture age had no effect. To explore whether culture age will alter *Dunaliella* mass spectra, a time series experiment was conducted using CCAP 19/27 and 19/7C obtaining mass spectra from a range of growth phases spanning exponential growth through to early senescence. The spectral pattern did not change for either isolate; the only difference was peak intensity (supplementary Fig. 2). Therefore, in the case of *Dunaliella* it would appear that the reproducibility of the spectra is independent of culture age. However, it should be noted that we did not determine whether growth media composition affected the spectra.

Biotyping by MALDI-TOF-MS has provided compelling evidence of much greater strain diversity than previously recognised within the CCAP *Dunaliella* collection, particularly where the shortcomings of DNA barcoding are evident. Taken together with DNA barcoding, biotyping by MALDI-TOF-MS should be considered as a part of a polyphasic approach to characterise microalgae and cyanobacteria. This information is not only of considerable value to the culture collection but also for those researchers that access and will eventually seek to exploit *Dunaliella* biodiversity for biotechnology goals.

## Methods

### Microalgae and cyanobacteria strains and culture conditions

Thirty two *Dunaliella* isolates (Table 1) and single strains of *Isochrysis galbana* (CCAP 927/1) and *Chlorella vulgaris* (CCAP 211/63) were obtained from the Culture Collection of Algae and Protozoa (CCAP; www.ccap.ac.uk). Single strains of *Tetraselmis suecica* and *Arthrospira platensis* (CCMP 1295) were obtained from Seasalter Shellfish Ltd (Kent, UK) and the Provasoli-Guillard National Centre for Marine Algae and Microbiota (NCMA; https://ncma.bigelow.org/) (formerly the Centre for Culture of Marine Phytoplankton).

All *Dunaliella* (except *D. acidophila*, CCAP 19/35) and *T. suecica* strains were batch cultured in 50 mL Erlenmeyer flasks with foam bungs using either sterile f/2 or double strength f/2 media^58^ following guidance on the CCAP website. Media were made with 1 μm filtered, UV sterilised and autoclaved natural seawater. *Dunaliella acidophila* was grown in AJS medium that was acidified to pH 1.5 using hydrochloric acid. *Isochrysis galbana* was cultured using f/2 supplemented with 1.06 × 10^−4^ M silicate, *A. platensis* was cultured using f/2 supplemented with 29.4 mM NaNO_3_ and adjusted to pH 8. *Chlorella vulgaris* was cultured in 250 mL Erlenmeyer flasks using Jaworski’s medium made with sterilised deionised water. Cultures were maintained within a temperature range of 18 ± 1 °C with a 16L:8D photoperiod. Lighting was provided by a combination of warm and cool fluorescent tubes giving a mean illuminance of 3000 lux. Culture optical densities (OD) were measured at 690 nm from 300 μL triplicate subsamples in a microplate using a FLUOstar OPTIMA fluorescence plate reader (BMG LABTECH). Prior to analysis the cultures were diluted into the OD range of 0.2-0.3 for consistency.

### Genomic DNA preparation and ITS gene sequencing

Genomic DNA extraction was carried out using the GenElute^TM^ Bacterial DNA Kit (Sigma Aldrich, UK) with the following modifications. A 1.8 mL aliquot of each *Dunaliella* culture was centrifuged at 14,000 *g*. The pellets were washed and resuspended in Lysis Buffer P (Stratec) and Proteinase K (Invisorb) and incubated at 65 °C for 30 minutes. The lysate was centrifuged to remove cell debris and then isolation continued as described in the manufacturer’s protocol. PCR reactions were conducted using the ITS4 (5’-TCCTCCGCTTATTGATATGC-3’) and ITS5 (5’-GGAAGTAAAAGTCGTAACAAG-3) primer pair (Sigma Aldrich, UK)^59^. Each reaction was in a total volume of 20 μL which contained 1x HF buffer, 0.5 μM of each primer, 200 μM of each dNTP, 15 μg BSA (Promega), 0.4 U Phusion DNA polymerase (ThermoScientific) and 1 μL of genomic DNA. The cycling conditions were 98 °C for 5 minutes followed by 30 cycles of 98 °C for 30 s, 42 °C for 30 s, 72 °C for 1 minute followed by a final extension step at 72 °C for 10 minutes. Pure PCR products (~782 bp) were cleaned up using the GenElute^TM^ PCR Clean-Up Kit (Sigma Aldrich, UK) according to the manufacturer’s instructions. PCR reactions that contained multiple PCR fragments were cleaned using QIAEX II Gel Extraction Kit (QIAGEN) according to the manufacturer’s instructions. Bidirectional DNA sequencing was performed by Geneius Laboratories Ltd (Cramlington, UK) using the same primers as for PCR. Chromatograms were checked using Chromas Lite (v2.1.1) and consensus sequences were constructed using the CAP3 sequence assembly programme^60^. The sequences were deposited in GenBank® with the respective accession numbers listed in Table 1.

### Sample preparation for MALDI-TOF analysis using whole cells

Twenty milligrams of the sample matrix (α-cyano-4-hydroxy-cinnamic acid; HCCA, Brucker Daltonics) was prepared by mixing 1 mL of 50% acetonitrile: 2.5% trifluoro-acetic acid (Sigma-Aldrich, UK). The matrix was vortexed and saturated by 30 minutes incubation at 25 °C in an ultrasonic water bath at 100% power (Grant Instruments, Cambridge) with a second vortex at 15 minutes. The matrix was then centrifuged at 14,000 g for 2 minutes (Sigma 1-15K microcentrifuge) and 50 μL aliquots prepared fresh for use. Preliminary work revealed that crude solvent based extracts of the algae isolates did not generate consistent high quality mass spectra (data not shown). In contrast, analysis using whole cells in water proved effective. One millilitre of each algae culture containing whole cells was centrifuged at 14,000 *g* for 5 minutes and the pellets washed twice with deionised water. Each pellet was then re-suspended in 50 μL of deionised water. Samples were mixed 1:1 with HCCA matrix and four 2 μL technical replicates were spotted on to a MTP 384 ground steel MALDI target plate (Bruker Daltonics) and air dried at room temperature for 20 minutes.

### MALDI-TOF parameters and data acquisition

Mass spectrometry was done using an UltraFlex II MALDI-TOF TOF (Bruker Daltonics GmbH, Leipzig, Germany) mass spectrometer with fuzzy control of laser intensity. Ion source 1 was set at 25 kV and ion source 2 was set at 23.5 kV with a laser frequency of 50.0 Hz, a detector gain of 1,650 V, and a gating maximum of 1,500 Da. Spectra were recorded in the positive linear mode for the mass range of 2,000 to 20,000 *m/z*. Each spectrum was obtained by averaging 600 laser shots acquired in the automatic mode. For data acquisition and validation measurements were performed in Auto Execute mode. The spectra were externally calibrated using the Bacterial Test Standard (Bruker Daltonics). The standard consisted of seven ribosomal proteins from *Escherichia coli* with added RNase A and myoglobin to cover a range of ca. 3637 to 16957 *m/z* (Da).

Four independent samples of each microalgae and cyanobacteria isolate were placed on four separate spots on a ground steel MALDI target plate (see supplementary Figure 3). Each sample spot was read twice thereby producing eight spectra per isolate. The quality and mass accuracy of the peaks were examined using the FlexAnalysis software (Bruker Daltonics). The eight spectra were overlaid and a consensus spectrum generated which was added to the database for each isolate.

### Time course growth study

To determine whether isolate spectra change with culture age the isolates CCAP 19/27 and CCAP 19/7C were batch cultured for 20 days using sterile f/2 medium in 500 mL Erlenmeyer flasks with foam bungs as described above. Cultures were aerated using compressed sterile-filtered air at 1.6 vessel volumes per minute. Cell counts were made using an improved Neubauer haemocytometer with an Olympus BH-2 brightfield microscope using Lugol’s solution as an immobilising agent. Samples that were collected on culture days 2, 9, 10, 12, 14, 18 and 20 were prepared for MALDI analysis as described above and the pellets stored at −80 °C. Prior to spotting, samples were thawed on ice and resuspended in 50 μL deionised water.

### Bacterial interference experiment

Two strains of *D. polymorpha* (CCAP 19/7A and 19/7C) were selected to investigate if bacterial mass spectra could potentially confound the spectra produced from the *Dunaliella* cultures. Two separate 150 mL cultures per isolate were grown in sterilized f/2 medium with one culture treated with the antibiotics penicillin G and dihydrostreptomycin sulphate in a ratio of 200 μg mL^−1^: 50 μg mL^−1^. One millilitre samples from each culture were inoculated onto triplicate sterile marine agar plates after four days of growth. The plates were incubated for eight days at 18 ^o^C and 16L:8D photoperiod. Whole bacterial colonies were spotted on to MALDI target plates and analysed as per the microalgae. Spectra from antibiotic and non-antibiotic treated *D. polymorpha* cultures were obtained for comparison.

### Data analysis

A phylogenetic tree was constructed for ITS data using Clustal Omega’s neighbour joining clustering method with a bootstrap value of 100 ( http://www.ebi.ac.uk/Tools/msa/clustalo/). Further ITS2 analysis was performed using the default settings of the University of Wuerzberg ITS2 workbench ( http://its2.bioapps.biozentrum.uni-wuerzburg.de/) and ProfDist for phylogenetic analysis using both sequence and structure information. For MALDI data a composite correlation index (CCI) distance matrix was constructed using the Biotyper software. From the distance matrix a heat map was generated in Microsoft Excel (Microsoft Corporation). The dendrogram was constructed by clustering of observations using Minitab (Minitab, Inc.) with the average linkage method.

## Additional Information

**How to cite this article**: Emami, K. *et al*. Proteomic-based biotyping reveals hidden diversity within a microalgae culture collection: An example using *Dunaliella*. *Sci. Rep.* **5**, 10036; doi: 10.1038/srep10036 (2015).

## Supplementary Material

Supplementary Information

## Figures and Tables

**Table 1:** The thirty two Culture Collection of Algae and Protozoa *Dunaliella* isolates used in this study including their respective ITS barcode accession numbers.

**Name assigned by depositor**	**CCAP strain no.**	**Origin**	**ITS rDNA GenBank accession**
*D. peircei*	CCAP 19/2	USA	KJ094608
*D. parva*	CCAP 19/9	England	KJ094607
	CCAP 19/10	Israel	KJ094617
	CCAP 19/1	Romania	KJ094618
	CCAP 19/26	Israel	KJ094630
*D. tertiolecta*	CCAP 19/6B	Norway	KJ094612
	CCAP 19/24	Unknown	KJ094628
	CCAP 19/27	Unknown	KJ094631
*D. quartolecta*	CCAP 19/8	England	KJ094616
*D. minuta*	CCAP 19/5	France	KJ094611
*D. bioculata*	CCAP 19/4	Soviet Union	KJ094610
*D. primolecta*	CCAP 19/34	England	KJ094636
*D. salina*	CCAP 19/3	Soviet Union	KJ094609
	CCAP 19/18	Australia	KJ094622
	CCAP 19/20	Unknown	KJ094624
	CCAP 19/25	Mexico	KJ094629
	CCAP 19/30	Israel	KJ094632
	CCAP 19/31	Unknown	KJ094633
	CCAP 19/39	Spain	KJ094637
*D. acidophila*	CCAP 19/35	Italy	KP005451
*D. polymorpha*	CCAP 19/7A	England	KJ094613
	CCAP 19/7B	England	KJ094614
	CCAP 19/7C	England	KJ094615
*Dunaliella* sp.	CCAP 19/22	Unknown	KJ094619
	CCAP 19/33	England	KJ094620
	CCAP 19/15	Israel	KJ094621
	CCAP 19/21	Egypt	KJ094623
	CCAP 19/23	Unknown	KJ094625
	CCAP 19/14	Israel	KJ094626
	CCAP 19/12	Israel	KJ094627
	CCAP 19/19	Australia	KJ094634
	CCAP 19/32	USA	KJ094635

**Table 2:** Presence (+) or absence (−) of nine major protein peaks (m/z up to ± 10 units) as identified by MALDI-TOF-MS from the eighteen *Dunaliella* isolates assigned to clade 2 in the ITS rDNA dendrogram (Fig. 1).

***m/z***	**3045**	**4627**	**5803**	**7588**	**8711**	**9254**	**10157**	**13019**	**18101**
CCAP 19/4	+	+	+	−	+	+	+	+	−
CCAP 19/5	+	4327	+	+	−	−	+	+	−
CCAP 19/6B	3091	+	5881	−	+	−	+	+	−
CCAP 19/7A	+	+	+	−	+	+	+	+	−
CCAP 19/7B	+	+	+	−	+	+	+	+	−
CCAP 19/7C	+	+	+	−	+	+	+	+	−
CCAP 19/8	+	+	+	−	+	+	+	+	−
CCAP 19/9	+	+	−	+	+	+	+	+	−
CCAP 19/12	+	+	+	+	+	−	+	+	−
CCAP 19/14	+	+	+	−	+	+	+	+	−
CCAP 19/22	+	+	+	−	+	+	+	+	−
CCAP 19/23	+	+	−	+	+	+	+	+	−
CCAP 19/24	+	+	+	−	+	+	+	+	−
CCAP 19/27	+	+	−	−	+	+	+	+	−
CCAP 19/31	+	+	−	+	+	+	+	+	−
CCAP 19/32	−	+	−	−	−	9278	+	+	−
CCAP 19/33	+	+	−	−	−	9266	+	−	−
CCAP 19/34	+	+	+	−	+	+	+	+	+

In cases where a similar peak with a m/z of more than 10 mass unit differences was observed the exact m/z values are indicated in the columns. These changes in peak mass would suggest potential oxidation and amino acid substitution respectively ( www.Unimod.org/). Some of the observed differences in the mass to charge ratios may be due to structural modifications such as amino acid oxidation, substitution or protein glycosylation; or uneven cell distribution due to the topology of the analyte/matrix mixture on the MALDI plate.
