# Supplementary material for: Proteomic-based biotyping reveals hidden diversity within a microalgae culture collection: An example using Dunaliella
Source: Sci Rep. 2015 May 12;5:10036. doi: 10.1038/srep10036 (PMC4650328; doi:10.1038/srep10036)
Supplement: Supplementary Information [file srep10036-s1.doc]

**Supplementary Information**

**Proteomic-based biotyping reveals hidden diversity within a microalgae culture collection: An example using *Dunaliella*.**

Kaveh Emami^1^, Ethan Hack^2^, Andrew Nelson^3^, Chelsea M. Brain^1#^, Fern M. Lyne^1^, Ehsan Mesbahi^4^, John G. Day^5^, Gary S. Caldwell*^1^

^1^ School of Marine Science and Technology, Newcastle University, Newcastle upon Tyne, NE1 7RU, UK.

^2^School of Biology, Newcastle University, Newcastle upon Tyne, NE1 7RU, UK.

^3^Faculty of Health and Life Sciences, Northumbria University, Newcastle upon Tyne, NE1 8ST.

^4^Faculty of Science Agriculture and Engineering (SAgE), Devonshire Building,
Newcastle University, Newcastle upon Tyne, NE1 7RU, UK.

^5^Culture Collection of Algae and Protozoa, Scottish Association for Marine Science, Scottish Marine Institute, Oban, Argyll, PA37 1QA, UK.

***** Corresponding Author: [gary.caldwell@ncl.ac.uk](mailto:gary.caldwell@ncl.ac.uk)

Tel.: +44 (0)191 208 6660; Fax.: +44 (0)191 208 7891

# Current address: Enterprise and Growth, University of Surrey, GU2 7XH, UK.


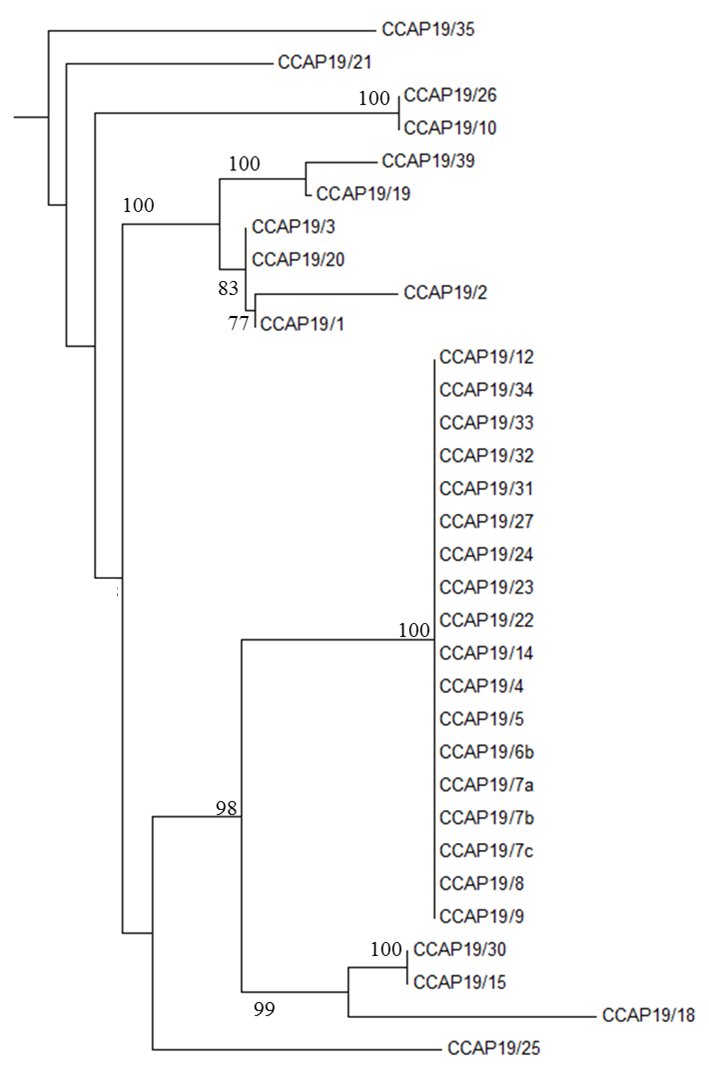


**Supplementary Figure 1.** ITS2 analysis using the default settings of the University of Wuerzberg ITS2 workbench and ProfDist for phylogenetic analysis using both sequence and structure information.


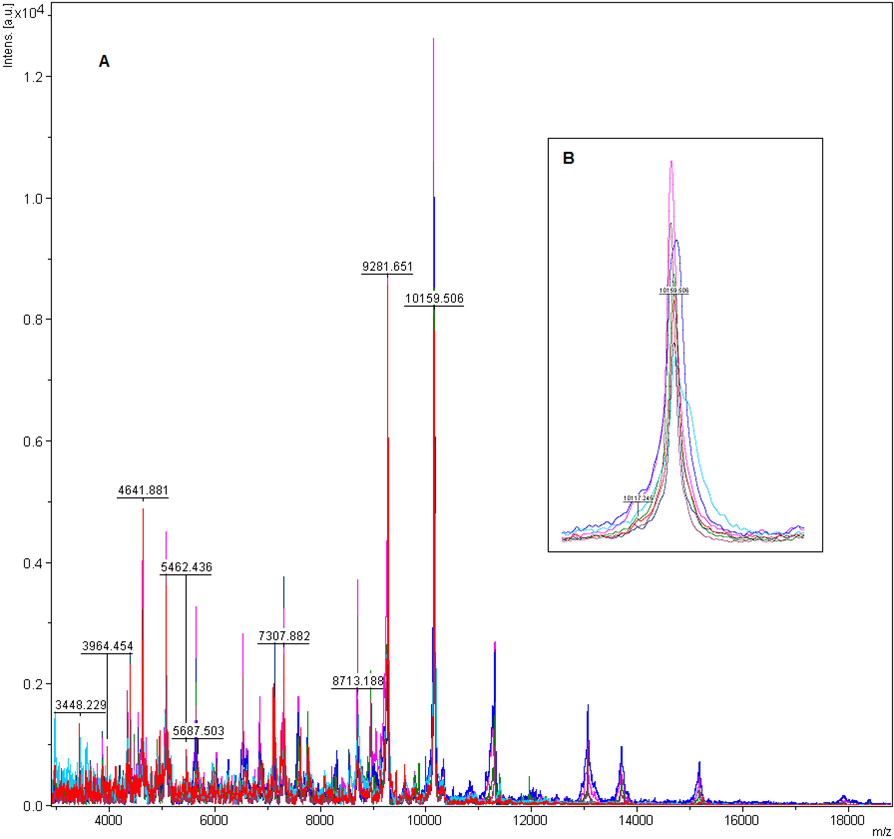


**Supplementary Figure 2.** MALDI-TOF mass spectra of CCAP 19/7C for seven sampling events between culture days 6-20. The samples were prepared in days 2, 9, 10, 12, 14, 18 and 20 after inoculation of the medium. The cultures were experiencing exponential growth on day 2, linear growth on days 9 and 10, early stationary phase on days 12 and 14 and in late stationary/early senescence on days 18 and 20. Although the intensity of the peaks varied, all peaks were overlapping and no extra or missing peaks were observed during the course of the experiment. Panel A presents the overall spectra between *m/z* 300-19000; Panel B is an example of the overlapped peak at *m/z* 10159. This isolate is representative of clade 2 as presented in Figure 1 in which nearly all isolates have major peaks at *m/z* 3045, 4627, 8711, 9254, and 10157 (± 2 *m/z*).


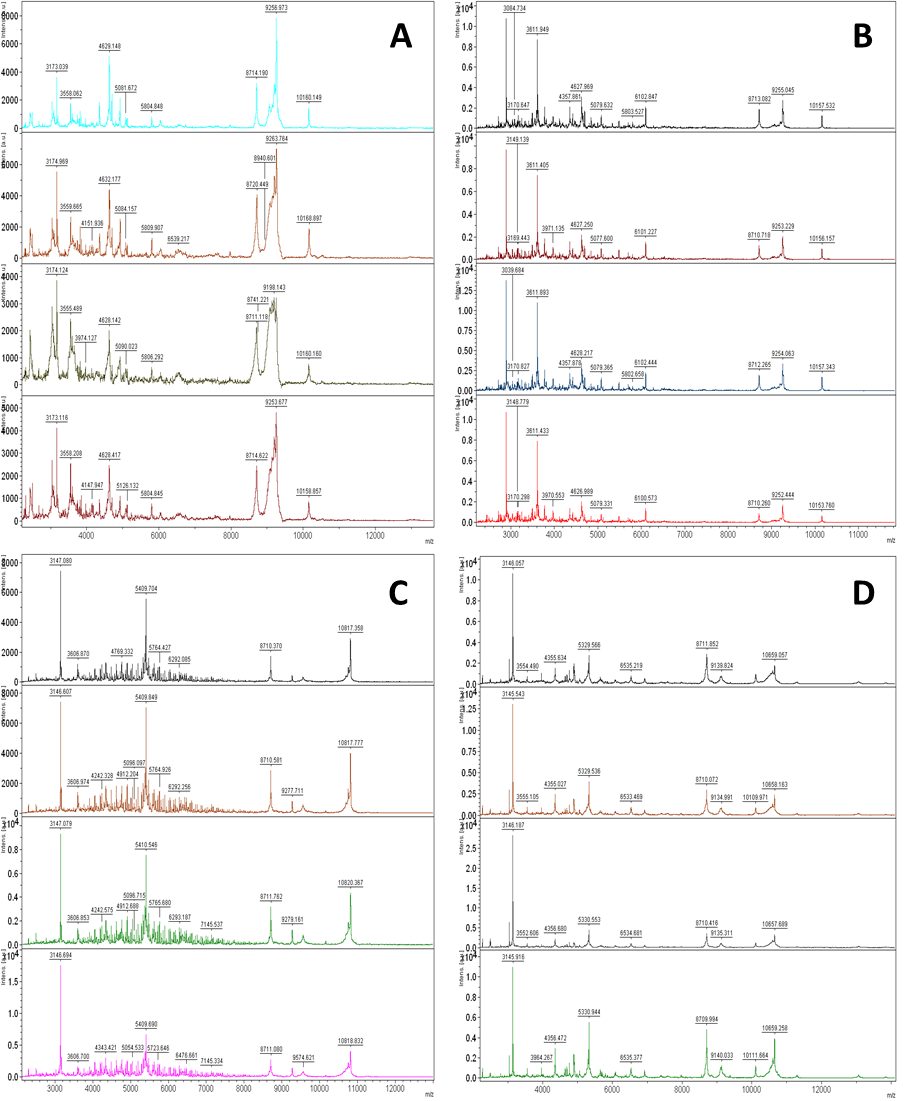


**Supplementary Figure 3.** Replicate (n = 4) MALDI-TOF mass spectra of four representative *Dunaliella* strains; A) CCAP 19/7A, B) 19/7C, C) 19/18 and D) 19/25, demonstrating the high degree of reproducibility within sample replicates.


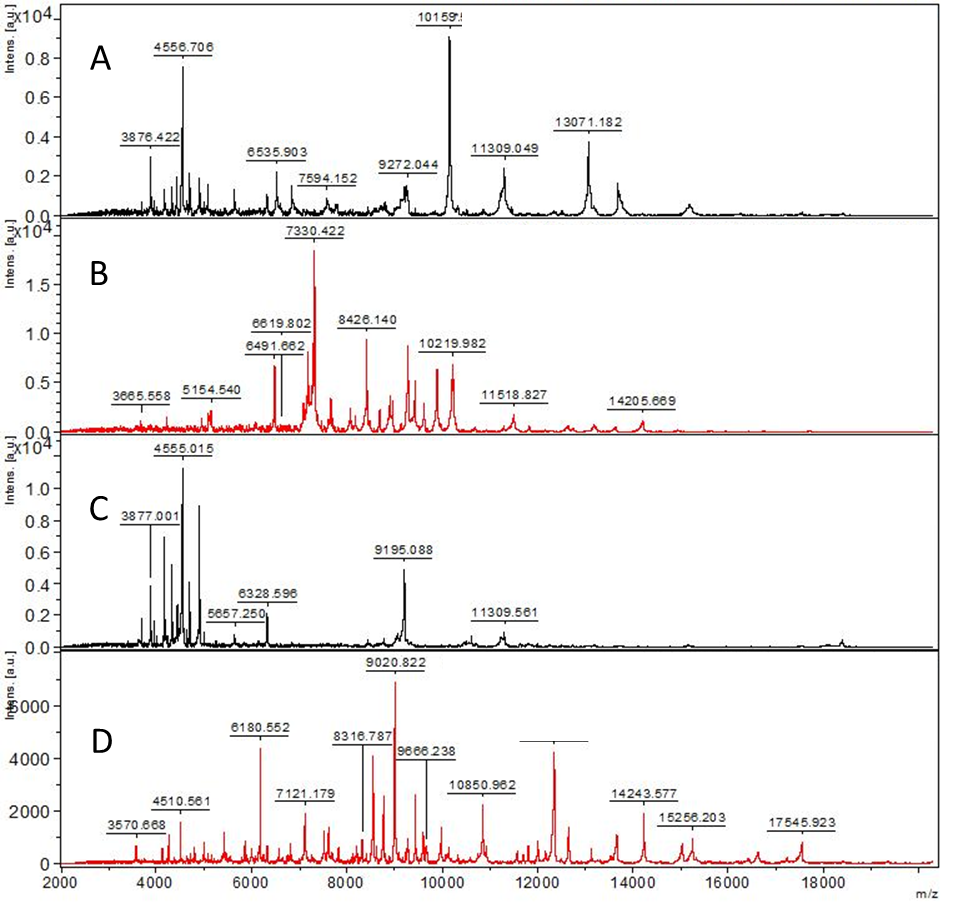


**Supplementary Figure 4.** MALDI-TOF mass spectra for *Dunaliella polymorpha* together with the spectra from the most numerically dominant bacteria within each culture. A) CCAP 19/7A; B) bacteria from CCAP 19/7A; C) CCAP 19/7C; and D) bacteria from CCAP 19/7C. There was no major overlap between the main peaks of the algae and the dominant bacterium from each culture.
